# Compared effects of inhibition and exogenous administration of hydrogen sulphide in ischaemia-reperfusion injury

**DOI:** 10.1186/cc12808

**Published:** 2013-07-10

**Authors:** Khodor Issa, Antoine Kimmoun, Solène Collin, Frederique Ganster, Sophie Fremont-Orlowski, Pierre Asfar, Paul-Michel Mertes, Bruno Levy

**Affiliations:** 1 CHU Nancy, Groupe Choc Inserm, U961, Faculté de Médecine, 54511 Vandoeuvre les Nancy, France; 2 Université de Lorraine, 54000 Nancy, France; 3 CHU Nancy, Service de Réanimation Médicale Brabois, Pole Cardiovasculaire et Réanimation Médicale, Hôpital Brabois, 54511 Vandoeuvre les Nancy, France; 4 Laboratoire HIFI UPRES EA 3859, Université d'Angers, Angers, France

## Abstract

**Introduction:**

Haemorrhagic shock is associated with an inflammatory response consecutive to ischaemia-reperfusion (I/R) that leads to cardiovascular failure and organ injury. The role of and the timing of administration of hydrogen sulphide (H_2_S) remain uncertain. Vascular effects of H_2_S are mainly mediated through K^+^_ATP_-channel activation. Herein, we compared the effects of D,L-propargylglycine (PAG), an inhibitor of H_2_S production, as well as sodium hydrosulphide (NaHS), an H_2_S donor, on haemodynamics, vascular reactivity and cellular pathways in a rat model of I/R. We also compared the haemodynamic effects of NaHS administered before and 10 minutes after reperfusion.

**Methods:**

Mechanically ventilated and instrumented rats were bled during 60 minutes in order to maintain mean arterial pressure at 40 ± 2 mmHg. Ten minutes prior to retransfusion, rats randomly received either an intravenous bolus of NaHS (0.2 mg/kg) or vehicle (0.9% NaCl) or PAG (50 mg/kg). PNU, a pore-forming receptor inhibitor of K^+^_ATP _channels, was used to assess the role of K^+^_ATP _channels.

**Results:**

Shock and I/R induced a decrease in mean arterial pressure, lactic acidosis and *ex vivo *vascular hyporeactivity, which were attenuated by NaHS administered before reperfusion and PNU but not by PAG and NaHS administered 10 minutes after reperfusion. NaHS also prevented aortic inducible nitric oxide synthase expression and nitric oxide production while increasing Akt and endothelial nitric oxide synthase phosphorylation. NaHS reduced JNK activity and p-P38/P38 activation, suggesting a decrease in endothelial cell activation without variation in ERK phosphorylation. PNU + NaHS increased mean arterial pressure when compared with NaHS or PNU alone, suggesting a dual effect of NaHS on vascular reactivity.

**Conclusion:**

NaHS when given before reperfusion protects against the effects of haemorrhage-induced I/R by acting primarily through a decrease in both proinflammatory cytokines and inducible nitric oxide synthase expression and an upregulation of the Akt/endothelial nitric oxide synthase pathway.

**Keywords**: hydrogen sulphide, inflammation mediators, therapeutic use, shock, hemorrhagic/drug therapy, haemodynamics/drug effects

## Introduction

The reperfusion phase of haemorrhagic shock is associated with an inflammatory response, including increased NF-κB activation [1], increased inflammatory cytokine production [2], increased nitric oxide (NO) production and inducible nitric oxide synthase (iNOS) gene expression [3,4], and increased activation of vascular K^+^_ATP _channels. These inflammatory responses are associated with hypotension, vasodilation and hyporesponsiveness to vasopressor agents and lead to ischaemia-reperfusion (I/R) organ injury [5]. Treating and/or preventing I/R-induced organ injury is therefore a major challenge.

Hydrogen sulphide (H_2_S) is recognised as a gasotransmitter, similar to NO and carbon monoxide. However, current knowledge relative to its role in physiology and pathology remains under discussion [6]. Many effects of H_2_S are the subject of controversy [7]. Depending on the chosen models, H_2_S has been reported to display opposite effects in haemorrhagic shock conditions. While inhaled H_2_S and intravenous sodium sulphide and sodium hydrosulphide (NaHS) reportedly increased survival [8], improved haemodynamics, attenuated metabolic failure in rodents [9-11], exerted cardioprotective effects [10,11] as well as protected against organ injury [12], sodium sulphide did not exert any beneficial effects in swine [13]. Moreover, in other studies, blocking H_2_S biosynthesis with D,L-propargylglycine (PAG), a cystathionine γ-lyase inhibitor, improved haemodynamics and attenuated systemic inflammation and organ injury [14,15].

The fact that H_2_S injection was associated with an increase in arterial pressure is intriguing. Currently available data indicate that H_2_S relaxes blood vessels [16] mostly, if not exclusively, by opening ATP-regulated potassium channels in vascular smooth muscle cells [17,18]. We hypothesised that H_2_S injected at the time of reperfusion could decrease the consequences of shock and reperfusion, that the use of an inhibitor of endogenous H_2_S production leads to opposite effects, and that adding a vascular K^+^_ATP_-channel inhibitor would improve the effects of H_2_S on systemic haemodynamics. Using a previously published model of I/R induced by haemorrhagic shock, we thus compared the effects of H_2_S and of its inhibition as well as of K^+^_ATP_-channel inhibition on haemodynamics, vascular reactivity and cellular pathways.

## Materials and methods

The study protocol was approved by the Nancy Institutional Committee on Animal Care and Use. The experiments were performed in conformity with the European legislation on the use of laboratory animals.

### Animals

Adult male Wistar rats, weighing 325 ± 15 g, were housed under 12-hour light/dark cycles in the animal facility of the University of Nancy 1 (France).

### Surgical procedure

Animals were anaesthetised with intraperitoneal pentobarbital (50 mg/kg body weight). Rats were placed on a homeothermic blanket system to maintain rectal temperature between 36.8 and 37.8°C for the duration of the experiment. After local anaesthesia with lidocaine 1% (AstraZeneca, Rueil-Malmaison, France**}**), a tracheotomy was performed and animals were mechanically ventilated (Harvard Rodent 683 ventilator; Harvard Instruments, South Natick, MA, USA) throughout the experiment. The ventilator was set to maintain carbon dioxide partial pressure in the vicinity of 40 mmHg and oxygen was added in order to maintain oxygen partial pressure above 100 mmHg. The left carotid artery was exposed and a 2.0 mm transit-time ultrasound flow probe (Transonic Systems Inc., Ithaca, NY, USA) was attached to the artery to continuously measure carotid blood flow (CBF).

Under local anaesthesia, the femoral artery was canulated in order to measure the mean arterial blood pressure (MAP) and heart rate (HR) on the one hand, and to induce haemorrhagic shock on the other. The homolateral femoral vein was canulated for retransfusion of withdrawn blood, for fluid replacement and for bolus infusion of either vehicle or drugs.

### Induction of haemorrhagic shock and protocol design

Surgery was followed by a 20-minute stabilisation period. Thereafter, haemorrhagic shock was induced by the graded withdrawal of blood from the femoral artery to a reservoir until MAP decreased to 40 mmHg and maintained during 60 minutes by further blood withdrawal or reinfusion of shed blood. At 60 minutes, shed blood was retransfused via the venous line within 10 minutes. Animals were continuously monitored for HR, MAP and CBF during 300 minutes. Hydration was performed with a perfusion of 0.9% NaCl at a rate of 1.2 ml/hour.

At the end of the experiment, rats were sacrificed and blood samples were collected for arterial lactate measurement, centrifuged (4,000 rpm, 15 minutes, 4°C) and plasma aliquoted and stored at -80°C until biochemical analysis. Organs (aorta, heart and liver) were also collected and stored at -80°C until biochemical analyses.

### Pharmacological modulation

The dehydrated NaHS powder (anhydrous, 2 g; Alpha Aesar GmbH & Co, Ward Hill, MA, USA) was dissolved in isotonic saline under argon gas bubbling until a concentration of 40 mM was obtained and intravenously administered as a single bolus (0.2 mg/kg body weight) 10 minutes before retransfusion or 10 minutes after the end of retransfusion (late NaHS). PNU-37883A (guanidine; 4-morpholinecarboximidine-*N*-1-adamantyl-*N*'-cyclohexyl hydrochloride) (Sigma Aldrich, St Quentin Fallavier, France) was dissolved in a 1:1 mixture of dimethyl sulphoxide and intravenously administered as a bolus (1.5 mg/kg) followed by 1 mg/kg/hour. The inducible NO synthase inhibitor 1400W (Sigma Aldrich) was administered intraperitoneally (20 mg/kg) at T0.

### Study design

Eight groups of eight rats were studied, namely: sham rats, haemorrhagic shocked rats, shock + PAG (CSE inhibitor)-treated rats (50 mg/kg), shock + NaHS-treated rats, shock + late NaHS-treated rats, shock + PNU-37883A-treated rats, shock + PNU + NaHS-treated rats, and shock + 1400W-treated rats.

### Monitoring and measurements

Arterial blood gases were controlled after the stabilisation period, in order to establish mechanical ventilation. Measurements of blood gas and blood glucose were recorded at baseline (t = 0 minutes at the beginning of haemorrhagic shock) and at two critical periods, namely at the end of reperfusion (t = 70 minutes) and at the end of the experiment (t = 300 minutes). MAP, HR, CBF and rectal temperature were recorded at baseline and every 10 minutes thereafter during the observation period.

Lactate concentrations were determined using an automated blood gas analyser (ABL5 Radiometer; Neuilly-Plaisance, France).

### Biochemical analyses

Plasma levels of IL-6 and TNFα were measured in duplicate with the use of rat IL-6 and TNFα ELISA kits (Quantikine ELISA; R&D Systems Europe, LILLE, France) according to the manufacturer's instructions. Results were expressed as picograms of the measured cytokine per millitre of plasma.

### Measurement of nitrite/nitrate

NO_2_^- ^and NO_3_^- ^are the primary oxidised products of NO reacting with water, and therefore the total concentration of NO_2_^-^/NO_3_^- ^in plasma was used as an indicator of NO production *in vivo*. Briefly, the nitrate in the supernatant was first reduced to nitrite by incubation with nitrate reductase (10 U/ml) and NADPH (629.2 µg/ml) at room temperature for 30 minutes. Thereafter, total nitrite concentration in the samples was measured by Griess reaction following the addition of 100 µl Griess reagent to 100 µl sample in a 96-well plate with a flat transparent bottom. The optical density at 550 nm was measured by an ELISA microplate reader and normalised with the optical density at 550 nm of standard saline solutions.

### RNA extraction and quantitative RT-PCR

Primers for quantitative RT-PCR were obtained from Eurogentec (Angers, France). Total RNA extraction was carried out with the RNA Plus mini kit (Qiagen, Courtaboeuf Cedex, France) according to the manufacturer's instructions. Total RNA was reverse-transcribed to cDNA using the iScript One-Step RT-PCR Kit for Probes (Biorad, Marnes-la-Coquette, France). cDNA obtained from the RT reaction was subjected to quantitative PCR using iTaq Fast SYBR Green Supermix with ROX (Biorad, Marnes-la-Coquette, France). The primer and concentrations were optimised according to the manufacturer's guidelines. Expression of, Kir6.1 mRNA and SUR2B mRNA were measured using iTaq Fast SYBR Green Supermix (Biorad).

The PCR reaction parameters were as follows: incubation at 50°C for 2 minutes, incubation at 95°C for 10 minutes, and thereafter 40 denaturation cycles at 95°C for 15 seconds and annealing and extension at 60°C for 1 minute. Each sample was determined in duplicate. To determine the relative mRNA levels, a standard curve for each gene was created using RNA isolated from the haemorrhagic shock group. Isolated RNA was reverse-transcribed, and dilution series of cDNA ranging from 1 pg to 10 ng were subjected to real-time PCR. The obtained threshold cycle values were plotted against the dilution factor to create a standard curve. Relative mRNA levels in test samples were then calculated from the standard curve.

### Vascular reactivity

For *in vivo *determination, basal and maximal MAP values obtained after administration of 1 µg/kg bolus of norepinephrine were recorded in the sham, haemorrhagic shock, haemorrhagic shock + PNU and haemorrhagic shock + 1400W groups.

For *ex vivo *determination, aortic rings and small mesenteric arteries were carefully dissected and mounted on a wire myograph (Danish Myo Technology, Arhus, Denmark). The experiments were performed at 37°C in a physiological salt solution with the following composition: NaCl 119 mM; KCl 4.7 mM; NaHCO_3 _14.9 mM; MgSO_4_·7H_2_O 1.2 mM; CaCl_2 _2.5 mM; KH_2_PO_4 _1.18 mM; glucose 5.5 mM, continuously bubbled with 95% O_2 _and 5% CO_2_.

After an equilibration period (at least 20 minutes) under optimal passive tension, two successive contractions in response to the combination of KCl depolarisation (100 mM) and phenylephrine (PE) (10 µM) (Sigma-Aldrich) were used in order to test the maximal contractile capacity of the vessels. After a 20-minute washout period, concentration-response curves to PE were elicited by cumulative administration of this vasoconstrictor agonist (1 nM to 100 µM) in order to determine the same concentration producing an equal level of contraction in the different groups. To study endothelium-dependent relaxation, aortic rings with functional endothelium were precontracted with PE (1 µM) and then exposed to increasing incremental concentrations of acetylcholine (1 nM to 100 µM; Sigma, St Louis, MO, USA). The presence of functional endothelium was confirmed with acetylcholine (1 µM), which elicited a relaxation superior to 50%.

### Western blotting

Aorta and small mesenteric arteries (200 to 230 μm) were homogenised and lysed. Proteins (20 µg) were separated on 10% SDS-PAGE. Blots were probed with the following antibodies: anti-iNOS (BD Biosciences, San Jose, CA, USA), phosphorylated endothelial nitric oxide synthase (p-eNOS) (rabbit anti-rat eNOS, phosphorylated (ser1177); Cell Signaling Technology Saint Quentin Yvelines, France), phosphorylated-Akt (p-Akt) (rabbit anti-rat Akt, phosphorylated (ser473); Cell Signaling Technology), phospho-SAPK/JNK (mouse, anti-rat SAPK/JNK, phosphorylated (Thr183/Tyr185); Cell Signaling Technology), phospho-p38 mitogen-activated protein kinase (mouse, anti-rat p38 MAPK, phosphorylated (Thr180/Tyr182); Cell Signaling Technology), and phosphor-p44/42 MAPK (Erk1/2) (rabbit anti-rat p44/p42 MAPK, phosphorylated (Thr1202/Tyr204); Cell Signaling Technology). Proteins were transferred onto nitrocellulose membranes and probed with a monoclonal mouse anti-α-Tubulin antibody (Sigma-Aldrich).

Bound antibodies were detected with a secondary peroxidase-conjugated anti-mouse IgG (Promega, Madison, WI, USA). The blots were visualised using an enhanced chemiluminescence system (ECL Plus; Amersham, GE Healthcare Europe, Velizy-Villacoublay, France).

### Statistical analyses

Results are expressed as the median and interquartile range for *n *experiments (*n *representing the number of animals). Difference between groups was tested using a Kruskal-Wallis test. When the relevant *F *values were significant at the 5% level, further pairwise comparisons were performed using a Dunn's multiple comparison test. All statistics were performed with the Statview software (version 5.0 software; SAS Institute, Cary, NC, USA). *P *<0.05 was considered statistically significant.

## Results

### Model characterisation

#### Shock and I/R-induced hypotension, lactic acidosis and vascular hyporeactivity to norepinephrine

**Figure 1:**
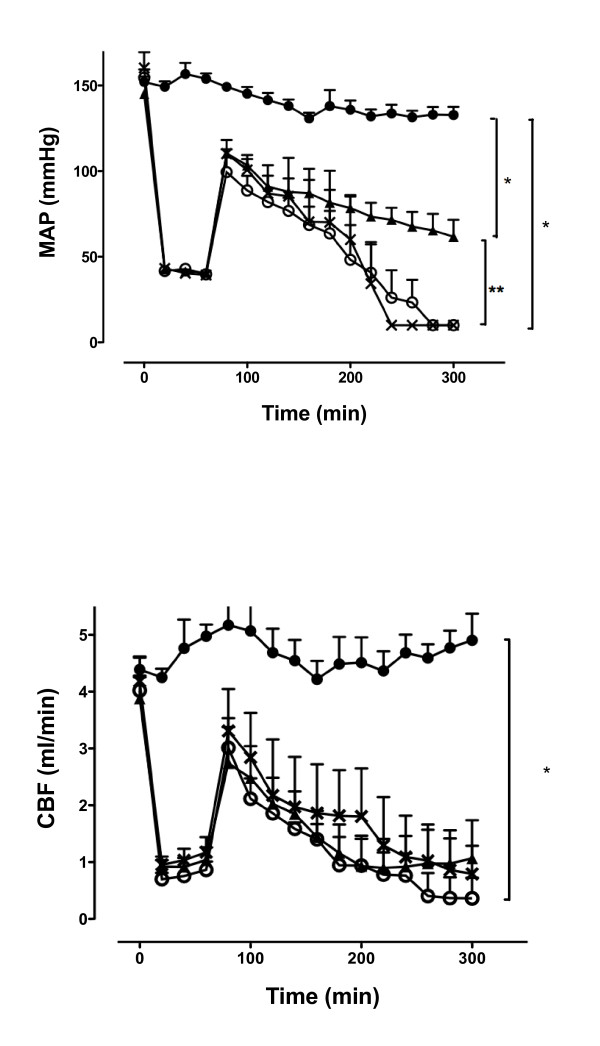
**Hemodynamic measurements**. **(A) **Mean arterial blood pressure (MAP) and **(B) **carotid blood flow (CBF) in the sham (filled circles), haemorrhagic shock + saline (crosses), haemorrhagic shock + sodium hydrosulphide (NaHS; triangles) and haemorrhagic shock + D,L-propargylglycine (PAG; empty circles) groups recorded during a 300-minute monitoring period. *P <0.05, significantly different from sham. **P <0.05 versus haemorrhagic shock + NaHS group.

**Figure 2:**
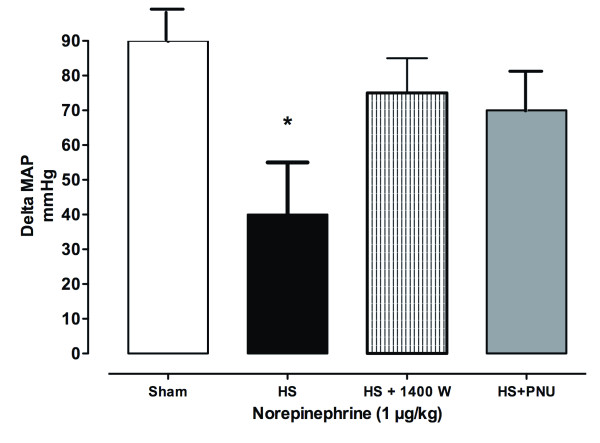
**Mean arterial pressure after administration of norepinephrine**. Mean arterial pressure (MAP) after administration of a bolus of 1 µg/kg norepinephrine in the haemorrhagic shock (HS) + saline, HS + 1400W (treated intraperitoneally with 1400W) and HS + PNU (treated with a 1-hour infusion of PNU-37883A; 1.5 mg/kg bolus followed by 1 mg/kg/hour) groups. *P <0.05, significantly different between HS + saline and all groups.

The HR, MAP and CBF remained stable throughout the experiment in the control group (Figure 1; see Additional file 1). In animals subjected to haemorrhagic shock and retransfusion, blood withdrawal significantly decreased the MAP, HR and CBF (Figure 1; see Additional file 1). Haemorrhagic shock was associated with a marked elevation in plasma lactate (9 × 2 mmol/l) compared with the sham group (2.1 × 0.5 mmol/l) (see Additional file 2), while the increase in arterial pressure induced by a bolus of 1 µg/kg norepinephrine was significantly decreased (*P *<0.01) in the shock group compared with sham animals (Figure 2).

#### Ischaemia-reperfusion is associated with overexpression/activation of iNOS and vascular K^+^_ATP _and increased proinflammatory cytokines

**Figure 3:**
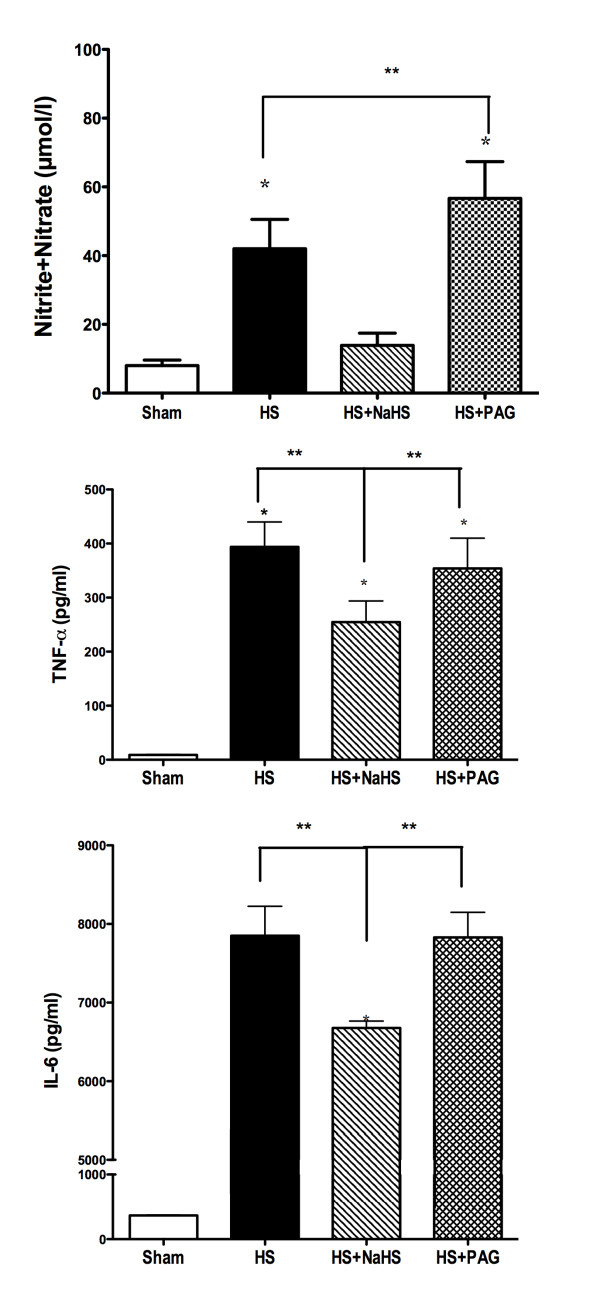
**Effects of sodium hydrosulphide and d,l-propargylglycine**. Effects of sodium hydrosulphide (NaHS) and D,L-propargylglycine (PAG) (50 mg/kg) on plasma levels of **(A) **nitrite + nitrate, **(B) **TNFα and **(C) **IL-6. Horizontal axes show the various groups. *P <0.05, significantly different between sham and all groups. **P <0.05 versus haemorrhagic shock (HS) + NaHS group.

IR-induced vascular hyporeactivity to a bolus of 1 µg/kg norepinephrine was completely restored following the administration of 1400W, a selective inhibitor of iNOS, as well as PNU-37883A, a pore-forming receptor inhibitor of K^+^_ATP _channels (Figure 2). I/R was associated with an increase in aortic and mesenteric protein expression Kir6.1 and SUR2B (Table 1). Plasma nitrite/nitrate (NO*_x_*), TNFα and IL-6 were also increased in shock-only rats (*P *<0.05) (Figure 3).

**Table 1:** mRNA expression of Kir6

		Haemorrhagic shock
Kir6.1	Aorta	21 × 5*
	Mesenteric	7 × 2*
SUR2B	Aorta	12 × 7*
	Mesenteric	3 × 0.3*

*n *= 7 in each group. *P < 0.05, significantly different between haemorrhagic shock and sham groups.

### Hemodynamic effects of NaHS administered 10 minutes after the end of reperfusion

**Figure 4:**
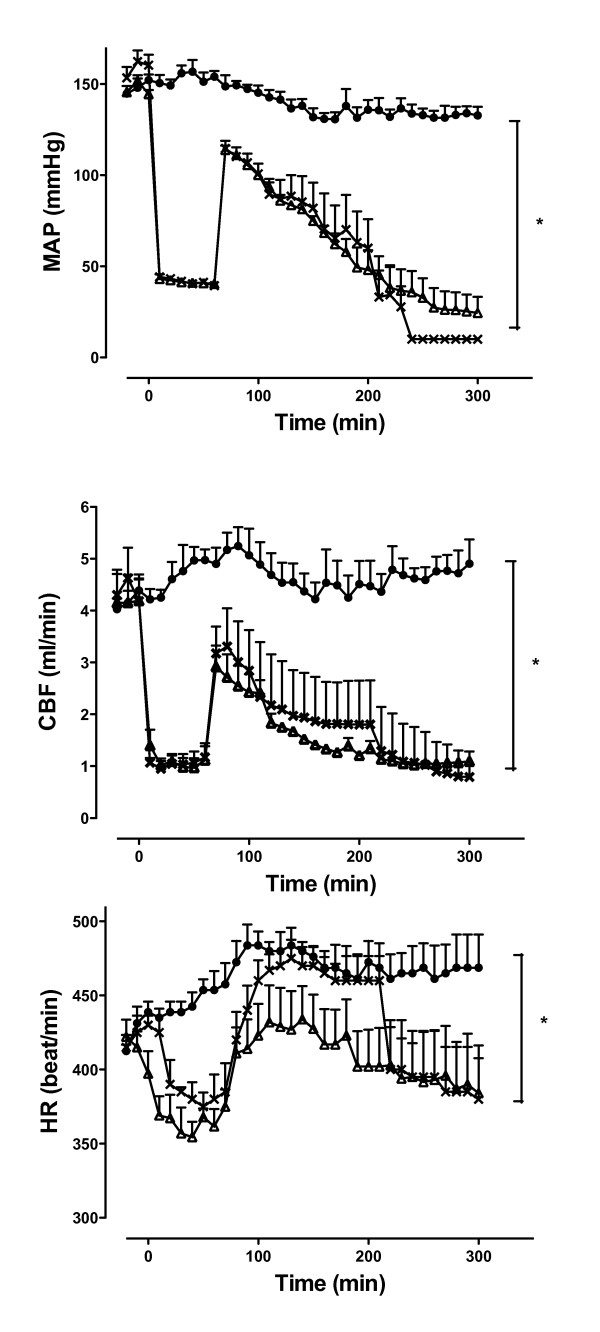
**Hemodynamic measurements with sodium hydrosulphide administered 10 minutes after the end of reperfusion**. Mean arterial blood pressure (MAP), carotid blood flow (CBF) and heart rate (HR) in sham (filled circles), haemorrhagic shock + saline (crosses), and haemorrhagic shock + sodium hydrosulphide (NaHS) (triangles) groups recorded during a 300-minute monitoring period. *P <0.05, significantly between sham.

MAP, HR and CBF were not different when compared between the late NaHS group and animals subjected to haemorrhagic shock and retransfusion (Figure 4).

### Comparative effects of NaHS and PAG

#### Hydrogen sulphide donor NaHS prevents I/R-induced hemodynamic and metabolic dysfunction while PAG, an inhibitor of endogenous H_2_S production, has no effects

NaHS but not PAG significantly attenuated the drop in MAP induced by I/R (*P *<0.05) (Figure 1) while CBF and HR (data not shown) remained unaffected (Figure 1). All animals treated with NaHS survived the haemorrhagic shock, while haemorrhagic shocked rats and PAG-treated rats had MAP < 40 mmHg (which we considered equivalent to death) at the end of the experiment. Haemorrhagic shock-induced hyperlactataemia was attenuated by NaHS (HS-NaHS 5 × 2.3 mmol/l) (*P *<0.05) but was not modified with PAG *(P <*0.05) (see Additional file 2). Compared with shock-only rats and shock + PAG rats, NaHS-treated animals had a significantly improved pH (*P *<0.05) at the end of the experiment (T_150_) (see Additional file 2).

#### Sodium hydrosulphide improves vascular function in rat aortic and small mesenteric vessels

**Figure 5:**
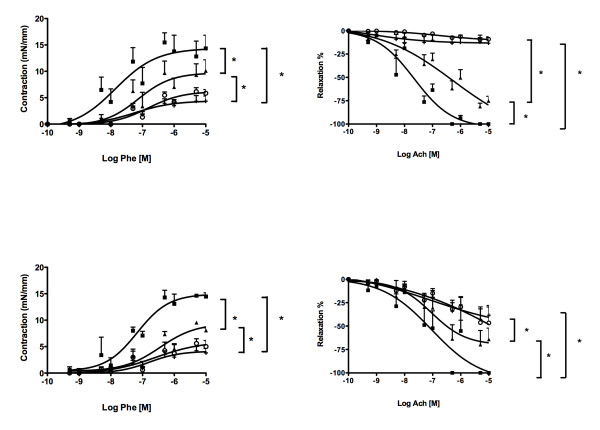
**Effects of treatment on phenylephrine-induced contraction in aorta and on aortic dilatation to acetylcholine**. Effects of treatment **(A) **on phenylephrine (Phe)-induced contraction in aorta and **(B) **on aortic dilatation to acetylcholine (ACh) in sham (filled squares), haemorrhagic shock + saline (crosses), haemorrhagic shock + sodium hydrosulphide (triangles) and haemorrhagic shock + D,L-propargylglycine (empty circles) groups. *P <0.05.

PE induced a dose-dependent increase in tension in aortic and small mesenteric vessels in control rats. In contrast, haemorrhagic shock blunted PE-stimulated contraction (*P *<0.01), whereas NaHS significantly restored the maximal contractile capacity to control levels (*P *<0.05) while PAG had no effect (Figure 5A). Acetylcholine produced a concentration-dependent relaxation of isolated aortic and small mesenteric vessels. Compared with the sham group, vascular responses to acetylcholine decreased in the aorta of shock-only rats (*P *<0.05). The addition of NaHS improved vascular response to acetylcholine while the inhibitor PAG did not modify endothelial function (Figure 5B).

#### Effect of NaHS on inflammatory mediators in haemorrhagic shock rats

Plasma nitrite/nitrate (NO*_x_*), TNFα and IL-6, which were increased in shock-only, control rats (*P *<0.05), decreased in NaHS-treated rats (*P *<0.05) and increased in PAG-treated rats (*P *<0.05) (Figure 3).

#### NaHS restores the phosphorylated Akt-to-Akt ratio and phosphorylated eNOS-to-eNOS ratio, while reducing haemorrhagic shock-induced upregulation of iNOS expression

**Figure 6:**
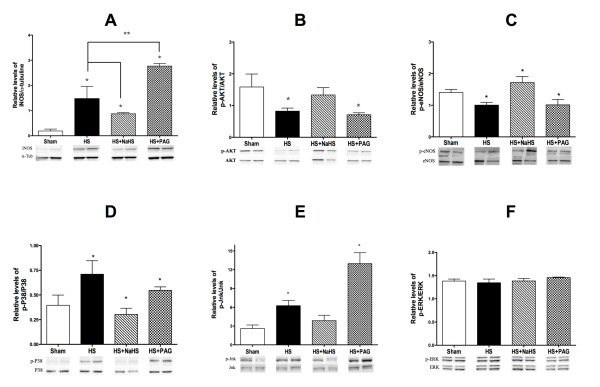
**Western blot analysis of protein expression**. Western blots revealing **(A) **inducible nitric oxide synthase (iNOS), **(B) **p-AKT, **(C) **phosphorylated endothelial nitric oxide synthase (p-eNOS), **(D) **p-p38, **(E) **p-JNK and **(F) **p-ERK expression. Proteins are expressed in whole lysates of aorta (*n *= 8) from all groups of rats. A typical western blot is shown below each histogram. Densitometric analysis was used to calculate the normalised protein ratio (protein to α-tubulin), which was set at 1 for the control group. Data are expressed as mean ± standard deviation. *P <0.05, significantly different versus sham and all groups. **P <0.05 versus haemorrhagic shock (HS) + sodium hydrosulphide (NaHS) group. PAG, D,L-propargylglycine.

Expression levels of Akt and phosphorylated Akt (Akt Ser473 phosphorylation) as well as phosphorylated Akt-to-Akt ratio were decreased in the aorta of shock-only rats (Figure 6). NaHS treatment blunted this decrease while PAG rather increased their expression levels (*P *<0.05). Similar results were also found for phosphorylated eNOS-to-eNOS ratio

The expression of iNOS protein, as assessed by western blotting, increased in shock-only rats (compared with rats from the sham group). This increase in iNOS expression was significantly reduced following the administration of NaHS (*P *<0.05) but increased with PAG (*P *p<0.05).

#### Effect of NaHS on alterations in p38 MAPK and JNK1/2 phosphorylation induced by haemorrhagic shock

NaHS reduced the phosphorylation of both p38 and JNK (Figure 6D,E). Conversely, PAG increased this phosphorylation compared with NaHS. Neither PAG nor NaHS influenced the phosphorylation of ERK (Figure 6F).

#### PNU-37883A, a pore-forming receptor inhibitor of K^+^_ATP _channels, further increases the effects of NaHS

PNU-NaHS was associated with a further increase in MAP when compared with NaHS alone (*P *<0.05) (see Additional file 3). PNU alone did not modify arterial pH nor the lactate level, whereas PNU-NaHS was associated with a decrease in lactate level and an increase in arterial pH as opposed to no differences with NaHS alone (*P *<0.05) (data not shown).

## Discussion

Herein, we illustrate the major role of NaHS in protecting the body against the consequences of shock and I/R [19]. Our findings revealed that the pharmacological inhibition of the endogenous pathway of H_2_S production during global I/R following a severe and reperfused haemorrhagic shock did not improve or worsen the consequences of shock, suggesting that endogenous H_2_S production *per se *is an active protective mechanism during IR; and we confirm that NaHS, an exogenous donor of H_2_S, is beneficial in terms of haemodynamics, tissue oxygenation and vascular reactivity. The effects of NaHS appear to be associated with a decrease in proinflammatory cytokines and a reduced expression of iNOS concomitant with a restoration of the eNOS pathway. These beneficial effects of NaHS appear to be more related to anti-inflammatory effects rather than to any specific vascular effect secondary to vascular K^+^_ATP _activation since selective inhibition of vascular K^+^_ATP _channels further improved haemodynamics and lactate metabolism in NaHS-treated rats. Furthermore, the effects of NaHS were not due to NaHS-induced hibernation since the animal's body temperature was continuously maintained. Finally, H_2_S when given after reperfusion was not efficient.

Our model was characterised by profound and ultimately lethal hypotension, decreased blood flow, lactic acidosis and vascular hyporesponsiveness to vasopressor agents. These haemodynamic disturbances were associated with iNOS upregulation, proinflammatory cytokine production and activation/upregulation of vascular K^+^_ATP _channels. The present findings confirmed that H_2_S given prior to retransfusion limited the I/R-induced decrease in MAP without changing carotid blood flow and heart rate when compared with shock-only rats. Given that H_2_S is usually considered an endogenous vasodilator acting through activation of vascular K^+^_ATP_, the role of this activation was further assessed with the selective vascular K^+^_ATP _blocker, PNU-37883A. Our results first demonstrated that K^+^_ATP _channels were overactivated and overexpressed both at the gene and protein levels in this model, indicating that vascular K^+^_ATP _is implicated in vascular hyporesponsiveness to vasopressor agents. Secondly, rats treated with H_2_S + PNU exhibited a higher mean arterial pressure and a better vasoreactivity to norepinephrine. This may explain why H_2_S, which is generally regarded as an endogenous vasodilator, paradoxically increased MAP in this model. H_2_S probably increases MAP through its well-demonstrated effects on the inflammatory pathway on the one hand, while decreasing MAP through K^+^_ATP _activation on the other, with the global result being an increase in MAP [20].

Potassium channels are critical metabolic sensors during acute metabolic changes such as hypoglycaemia or hyperglycaemia, ischaemia and hypoxia [21]. I/R-induced cardiovascular failure is traditionally ascribed to the effects of inflammatory mediators that induce circulatory changes with resulting tissue hypoxia and cell damage [22]. In the face of these deleterious signals, the body's adaptive response at the vascular level is to preserve cell survival through metabolic sensors by increasing local blood flow in the microcirculation, the so-called metabolic vasodilatation, in which the opening of K^+^_ATP _channels plays a major role [23]. This adaptive response also leads to systemic vasodilatation, hypotension and potentially multiple organ failure and death. Vascular potassium channels may thus have protective but also harmful roles during shock. Therefore, while the use of channel inhibitors might be an attractive option to counteract systemic vasodilatation, it may also act as a double-edged sword. Whether K^+^_ATP _activation is a protective phenomenon in this setting of disturbed microcirculation thus remains unknown.

### Hydrogen sulphide and PAG exert opposite effects on pathways implicated in vascular failure

Ganster and colleagues demonstrated that H_2_S improved cardiovascular status in I/R by decreasing oxidative stress and inflammation through a decrease in NF-κB activation [9]. Our present model was associated with an increase in proinflammatory and anti-inflammatory cytokines, an increase in iNOS expression and an alteration in eNOS phosphorylation. As for the phosphorylation pathway, JNK phosphorylation was increased without significant changes in the p-P38/P38 ratio. Indeed, JNK and P38 have been shown to be activated by TNF and IL-1 stimulation of endothelial cells [24] and to induce expression of proinflammatory effector molecules.

In the present study, H_2_S was found to decrease the cytokine storm as well as both gene and protein iNOS expression while increasing Akt and eNOS phosphorylation. Moreover, H_2_S reduced JNK activity and p-P38/P38 activation, suggesting a decrease in endothelial cell activation [25]. Conversely, all of these parameters were either not altered or worsened with PAG injection.

### Study limitations

The present model presents several limitations, first of which involves the use of a pressure-fixed and anaesthetised model of haemorrhagic shock that does not fully represent all of the specific patterns of human haemorrhagic shock.

Secondly, we used a fixed dose of NaHS that we previously found efficient without performing a dose-response study, thus leaving the possibility that potentially toxic or beneficial effects may have been missed.

Thirdly, we did not observe any differences between the shock group and the PAG-treated group with regard to haemodynamics, metabolism and proinflammatory cytokine parameters. Van de Louw and Haouzi recently demonstrated that, despite a severe cumulative oxygen debt (100 to 140 ml/kg), H_2_S blood and tissue concentrations did not change [26]. Nevertheless, despite the absence of a marked increase during H_2_S treatment, blocking endogenous H_2_S production most probably has little therapeutic benefit and may actually prove to be contraindicated [27].

Fourthly, when compared with mouse and humans, rats exhibited more iNOS activation during stress. The importance of the H_2_S-induced decrease in iNOS activation should therefore be discussed.

Lastly, the timing of H_2_S administration might be discussed. While pretreatment with inhaled H_2_S and intravenous sodium sulphide attenuated kidney, heart, and brain damage in mice undergoing I/R injury or cardiac arrest [28,29], similar post-treatment had no effect [12,30]. Our findings are in agreement with these previous reports suggesting that H_2_S beneficial effects seem to be confined to a narrow timing window.

## Conclusion

The present *in vivo *experimental study of I/R following resuscitated haemorrhagic shock in rats demonstrates that H_2_S administered exogenously before reperfusion is protective against the deleterious cardiovascular effects of haemorrhage-induced I/R. On the contrary, blocking endogenous H_2_S production or administering H_2_S after the reperfusion had no effect. More specifically, H_2_S decreases proinflammatory cytokine and iNOS expression and restores the Akt/eNOS pathway. Such beneficial effects of H_2_S donors warrant further experimental studies.

## Key messages

• H_2_S, administered exogenously before reperfusion is protective against the deleterious cardiovascular effects of haemorrhage-induced I/R.

• H_2_S is not effective when given after reperfusion.

• H_2_S increased MAP through anti-inflammatory effects despite vasodilatory effects due to K^+^_ATP_-channel activation.

• H_2_S decreases proinflammatory cytokine and iNOS expression and restores the Akt/eNOS pathway.

## Abbreviations

Akt: protein kinase B; CBF: carotid blood flow; ELISA: enzyme-linked immunosorbent assay; eNOS: endothelial nitric oxide synthase; ERK: extracellular signal-regulated kinases;H_2_S: hydrogen sulphide; HR: heart rate; IL: interleukin; iNOS: inducible nitric oxide synthase; I/R: ischaemia-reperfusion; JNK: c-Jun NH(2)-terminal protein kinases; K^+^_ATP_: ATP-regulated potassium channels; MAP: mean arterial pressure; MAPK: mitogen-activated protein kinase; NaHS: sodium hydrosulphide; NF: nuclear factor; NO: nitric oxide; PAG: D,L-propargylglycine; PCR: polymerase chain reaction; PE: phenylephrine; PNU: 4-morpholinecarboximidine-*N*-1-adamantyl-*N*'-cyclohexyl hydrochloride; p-P38/P38: phosphorylated/nonphosphorylated P38 kinase; RT: reverse transcriptase; TNF: tumour necrosis factor.

## Competing interests

The authors declare that they have no competing interests.

## Authors' contributions

KI participated in the study design, ran the experiments, performed the analysis and helped to draft the manuscript. SC and SF-O performed K^+^_ATP _RT-PCR and western blot. AK, FG, PA and P-MM participated in data analysis and helped to draft the manuscript. BL conceived the study and wrote the manuscript. All authors read and approved the final version of the manuscript.

## Supplementary Material

Additional file 1 Heart rate in sham (filled circles), haemorrhagic shock + saline (crosses), haemorrhagic shock + NaHS (triangles) and haemorrhagic shock + PAG (empty circles) groups recorded during a 300-minute monitoring period. *P < 0.05. Click here for file

Additional file 2 **Metabolic parameters. Evolution of (A) lactate and (B) pH**. *P <0.05, significantly different versus sham group. **P <0.05 versus haemorrhagic shock + NaHS group. Click here for file

Additional file 3 **Hemodynamic measurements**. MAP in the haemorrhagic shock + saline group (crosses), haemorrhagic shock + PNU (empty circles) group and haemorrhagic shock + PNU-NaHS (inversed triangles) rats recorded during a 300-minute monitoring period. *P <0.05. Click here for file
