# Supplementary figures and images for: Compared effects of inhibition and exogenous administration of hydrogen sulphide in ischaemia-reperfusion injury
Source: Crit Care. 2013 Jul 10;17(4):R129. doi: 10.1186/cc12808 (PMC4057116; doi:10.1186/cc12808)

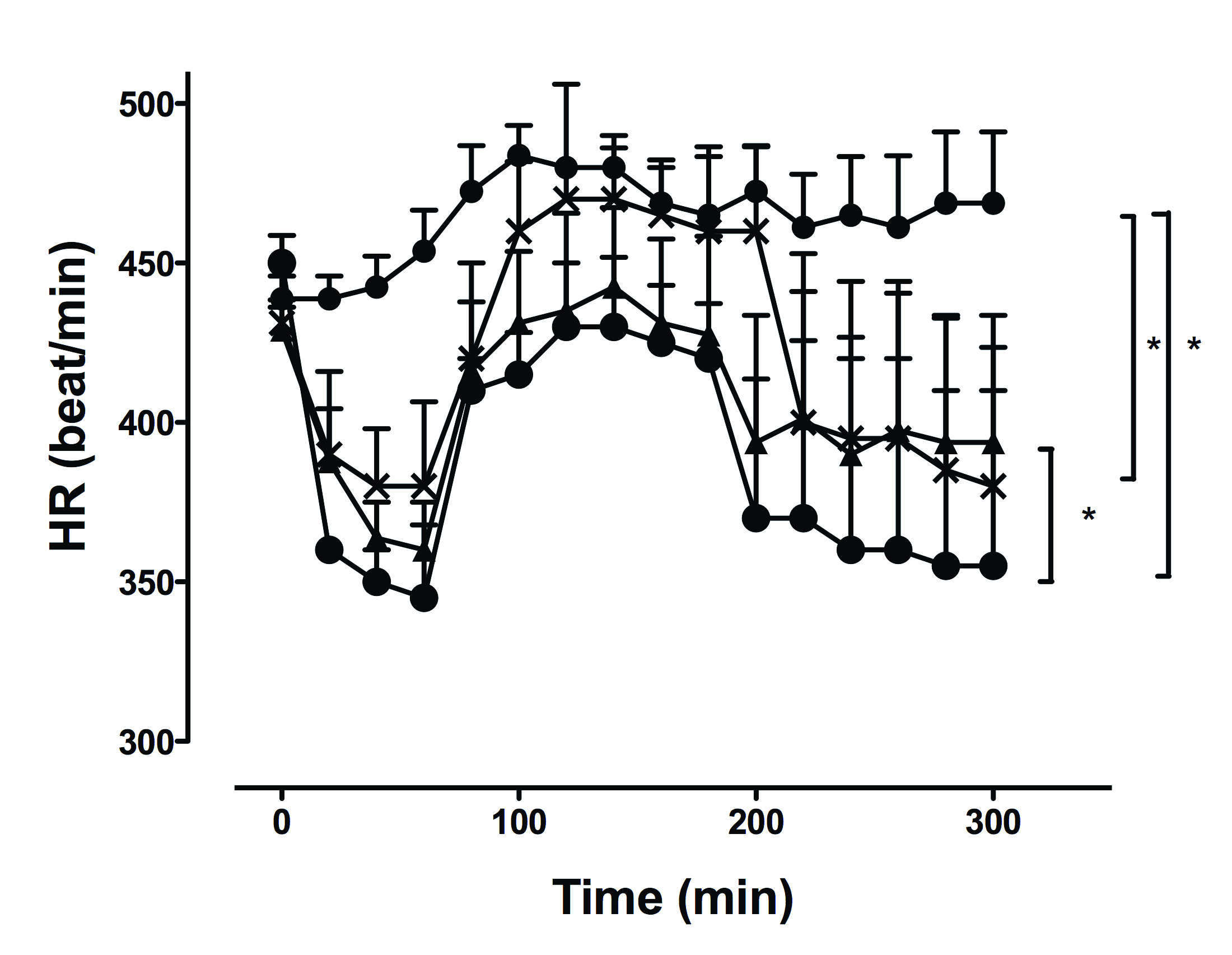

Supplement: Additional file 1 — Heart rate in sham (filled circles), haemorrhagic shock + saline (crosses), haemorrhagic shock + NaHS (triangles) and haemorrhagic shock + PAG (empty circles) groups recorded during a 300-minute monitoring period. *P < 0.05. [file cc12808-S1.TIFF]

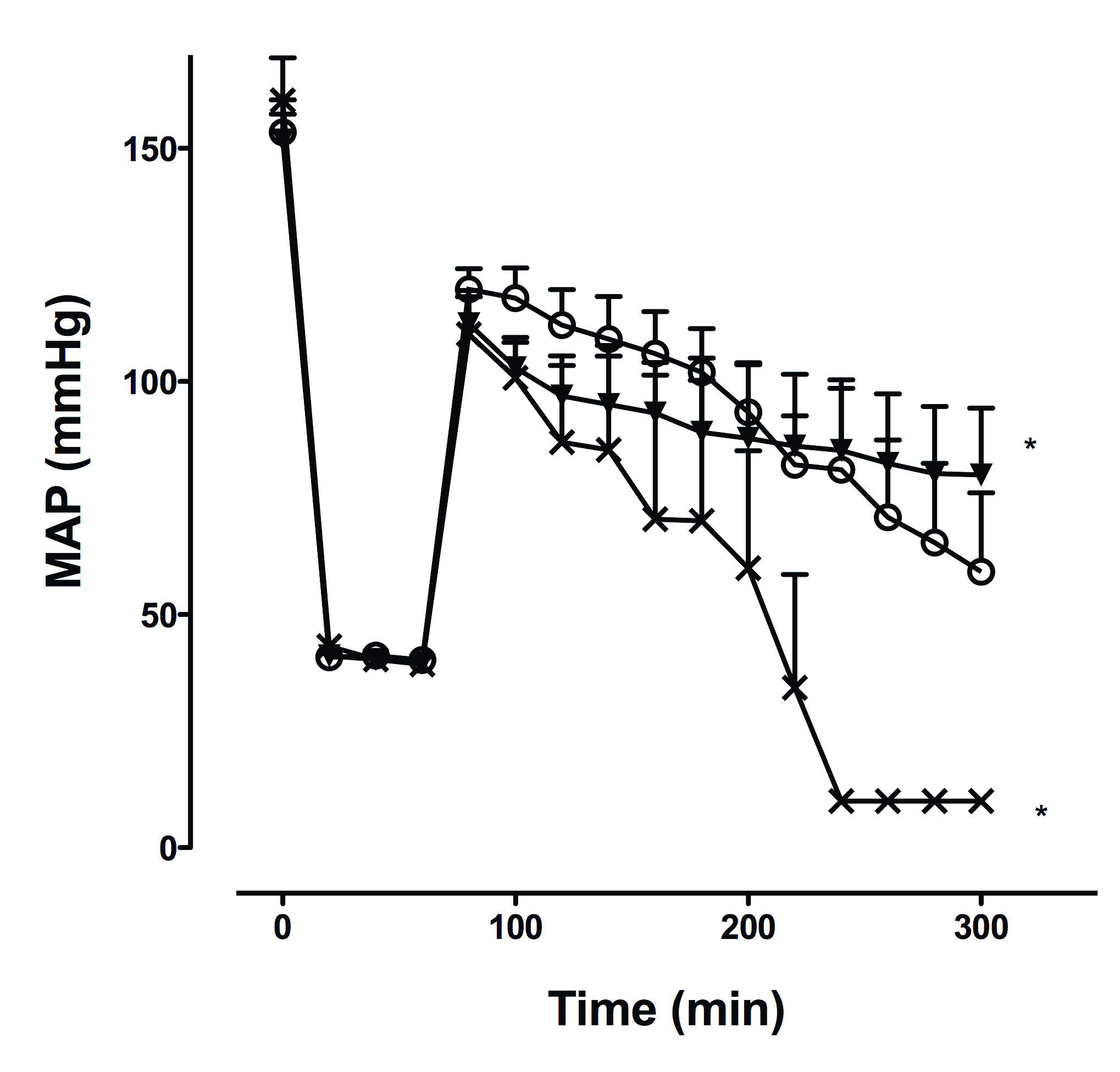

Supplement: Additional file 3 — Hemodynamic measurements. MAP in the haemorrhagic shock + saline group (crosses), haemorrhagic shock + PNU (empty circles) group and haemorrhagic shock + PNU-NaHS (inversed triangles) rats recorded during a 300-minute monitoring period. *P <0.05. [file cc12808-S3.TIFF]
